# Symbionts unmask the latent trade-off between reproduction and survival

**DOI:** 10.1016/j.isci.2026.116582

**Published:** 2026-07-01

**Authors:** Mariana Gabrielle Cangco Reyes, Tanzil G. Malik, Ke-An Hong, Syuan-Jyun Sun

**Affiliations:** 1 International Degree Program in Climate Change and Sustainable Development, National Taiwan University, Taipei 106319, Taiwan; 2 Earth Observation Centre, Institute of Climate Change, National University of Malaysia (UKM), Bangi, Selangor 43600, Malaysia

**Keywords:** ectosymbionts, host-parasite interaction, life history trade-off, terminal investment, cost of reproduction

## Abstract

Reproductive trade-offs are fundamental to life history evolution, yet their expression is often obscured by individual variation in quality. Symbionts represent a critical environmental factor that may alter these trade-offs, but their impact on the link between reproductive effort and somatic maintenance remains poorly understood. Using burying beetles (*Nicrophorus nepalensis*) and their phoretic mite (*Poecilochirus carabi*), with a split-brood design to control for genetic background, we show that mite presence shapes both the magnitude and success of reproductive attempts. Brief physical exposure to mites alone was sufficient to trigger a plastic shift toward higher fecundity, even when mites are subsequently removed, producing brood sizes matching those of permanently mite-associated females. Crucially, survival analysis revealed that mites unmasked a latent trade-off between reproduction and longevity. These findings demonstrate how symbiotic interaction can both induce anticipatory life history shifts and reveal reproductive costs that remain hidden.

## Highlights

• Brief mite contact alone triggers a shift to higher female reproductive effort
• Cue-exposed females escalate reproduction without the somatic cost of live mites
• Only persistent mite presence unmasks a latent reproduction-survival trade-off
• Body mass loss is the shared physiological threshold linking reproductive effort to mortality

## Graphical abstract

**Figure:**
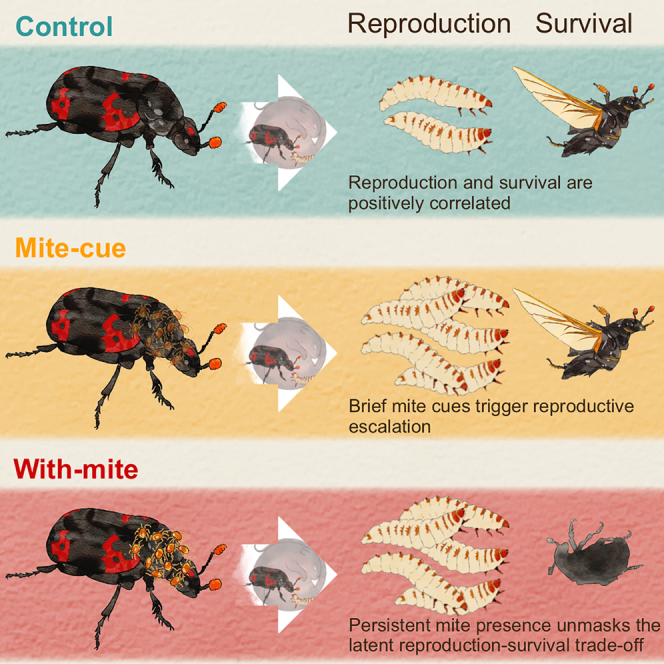

## Teaser

Biological sciences; Zoology; Evolutionary biology

## Introduction

Symbiotic interactions are fundamental components of ecological communities and play critical roles in shaping host behavior, life-history traits, and overall fitness.^1^^,^^2^ These interactions span a dynamic continuum from mutualism to parasitism, with outcomes that are often context-dependent, varying with environmental conditions and resource availability.^2^^,^^3^^,^^4^^,^^5^ Traditionally, the majority of research has emphasized consumptive effects, focusing on how symbionts, particularly parasites, alter host morphology and physiology through direct resource extraction or tissue damage.^6^^,^^7^^,^^8^ Under this framework, host costs are viewed primarily as a direct consequence of physical burden or infection pathology.

However, a growing body of evidence suggests that symbionts also influence hosts through subtle yet significant non-consumptive effects. Originally conceptualized in predator-prey ecology as risk effects,^9^ non-consumptive effects are defined as consequences arising from changes in host behavior, physiology, or life-history allocation driven by perceived risk rather than direct interaction costs. Crucially, non-consumptive effects can shape demography more markedly than direct predation.^10^ This framework can potentially be extended to host-parasite systems.^11^ Hosts may respond behaviorally or physiologically to cues indicating parasite presence even without direct infection or consumption.^12^^,^^13^^,^^14^ Just as tadpoles alter activity in response to parasite cues^15^ or mammals avoid parasite-rich foraging grounds,^16^ hosts frequently employ avoidance or heightened vigilance as first-line defenses. While these responses limit infection risk, they may impose substantial indirect costs by diverting energy away from somatic maintenance or reproduction.

Despite the recognition of non-consumptive effects, a critical gap remains in linking these cue-driven responses to life-history evolution.^17^ Most empirical studies have focused on immediate behavioral adjustments (e.g., avoidance or foraging shifts); far less is known about how the perceived presence of symbionts independently alters reproductive strategies and investment decisions. Does the perception of parasite risk trigger a shift in the trade-off between current reproduction and somatic maintenance, and does it do so by altering the threshold for initiating reproduction, the magnitude of investment once reproduction has begun, or the willingness to sustain investment through to offspring independence? Disentangling these cue-driven life-history shifts from the direct physiological drain of infection is essential for understanding the underlying evolutionary costs of symbiosis. While laboratory studies increasingly document significant physiological and fitness consequences of parasite exposure independent of infection status,^18^^,^^19^ the cumulative impact of these hidden costs on host populations and ecosystems remains widely underestimated.^13^^,^^20^^,^^21^

Burying beetles (*Nicrophorus* spp.) and their phoretic mites provide an ideal yet understudied system for investigating these dynamics.^22^^,^^23^^,^^24^ The beetles exhibit elaborate biparental care on small vertebrate carcasses, which serve both as breeding sites and contested resources vulnerable to microbial decay and interspecific competition.^25^^,^^26^ Phoretic mites, such as the widespread *Poechilochirus carabi*, attach to beetles for transport to fresh carcasses.^27^ While this phoretic stage is typically considered commensal due to minimal direct harm to the host,^23^^,^^28^ the relationship shifts dramatically upon carcass discovery. Once the mites disembark and reproduce alongside the hosts, they can potentially play a dualistic role: they can act as mutualists by suppressing competing blowfly larvae,^29^ but also exhibit context-dependent costs by directly consuming beetle eggs and larvae in the absence of interspecific competitors,^24^^,^^30^ or imposing cryptic physiological costs on adult beetles.^31^ Crucially, the brief sensory contact during phoresy itself may carry information value to the host: A high mite load on arrival could plausibly signal both a competitor-rich environment and a viable, freshly available carcass.^32^ Despite recognition of these dual roles, the extent to which beetle reproductive outcomes are driven by the direct burden of mites versus the perceived risk or informational value of their presence remains unresolved.^30^^,^^33^^,^^34^

**Figure 1:**
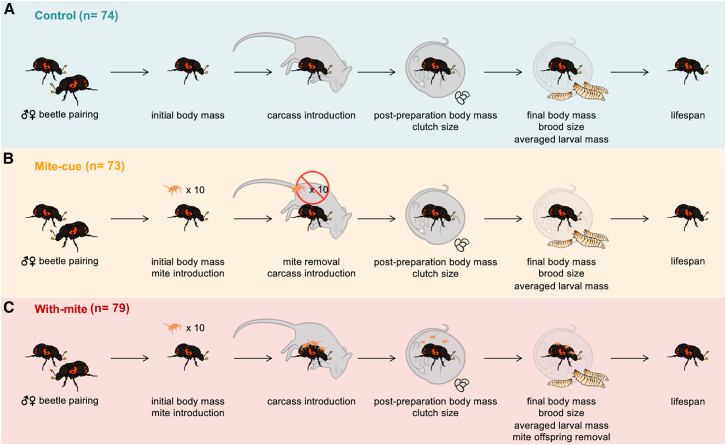
Experimental design and timeline A split-brood design was used where genetically standardized mating blocks were created by pairing brothers from a specific male lineage with sisters from a specific unrelated female lineage. These matched pairs were then distributed across the three treatments: (A) control, (B) mite-cue, and (C) with-mite. Illustration created by Megan M.Y. Chang.

In this study, we explicitly disentangle the direct and indirect, non-consumptive effects of mite presence on burying beetle reproduction and survival. We employed a manipulative experimental design where mites attach briefly to females but are removed prior to introduction to the carcass, isolating non-consumptive cue exposure from direct consumptive interactions during breeding. We quantified reproductive outcomes (clutch size, brood size, offspring quality), physiological costs (body mass dynamics), and residual fitness consequences (lifespan) (Figure 1). Guided by life-history theory and, in particular, the terminal investment hypothesis, which posits that perceived threats to future reproductive opportunities should favor increased current reproductive effort even at the expense of somatic maintenance and longevity.^35^ Specifically, we predicted that (1) mite-cue females would increase reproductive investment (larger broods) relative to controls, reflecting a risk- or carcass viability-induced shift toward current reproduction; (2) with-mite females would show similar or greater fecundity but at a steeper physiological cost, reflected in greater body mass loss during breeding; and (3) this body mass cost would translate into reduced post-reproductive survival in with-mite females, while mite-cue females, buffered from direct consumption, would maintain better body condition and longevity despite elevated reproductive effort. We additionally examined whether mite-associated cues influenced not only the magnitude of reproductive investment but also the probability of completing a breeding attempt, since cues that signal carcass viability may sustain maternal commitment through the larval stage even when they do not alter the initial decision to lay eggs.

By combining comprehensive metrics of reproduction, physiology, and survival within a facultative host-symbiont system, our study provides one of the first empirical tests of symbiont-induced non-consumptive effects on host life history. Ultimately, our findings illuminate how perceived risk and physiological cost interact to shape host reproductive strategies and reveal that even brief, non-consumptive effects of a symbiont can fundamentally reshape the trade-off between current reproduction and future survival.

## Results

### Mite exposure induces a shift in reproductive investment

Across all three treatment groups, the majority of females produced eggs, with rates ranging from 68.4% to 73.0% (control: 54/74, 73.0%; mite-cue: 52/73, 71.2%; with-mite: 54/79, 68.4%). There was no significant difference in the probability of egg production across the treatments (Chi-square test: X^2^ = 0.40, d.f. = 2, *p* = 0.817). However, the probability of breeding success varied across the treatments (control: 32/74, 43.2%; mite-cue: 34/73, 46.6%; with-mite: 43/79, 54.4%; Pearson’s Chi-squared test: X^2^ = 7.58, d.f. = 2, *p* = 0.023). Pairwise Fisher’s exact tests with Bonferroni correction revealed a significant difference in larval success between the control and with-mite treatments (adj. *p* = 0.031), while no significant differences were found between the control and mite-cue treatments (adj. *p* = 1.000) or the with-mite and mite-cue treatments (adj. *p* = 0.137).

**Figure 2:**
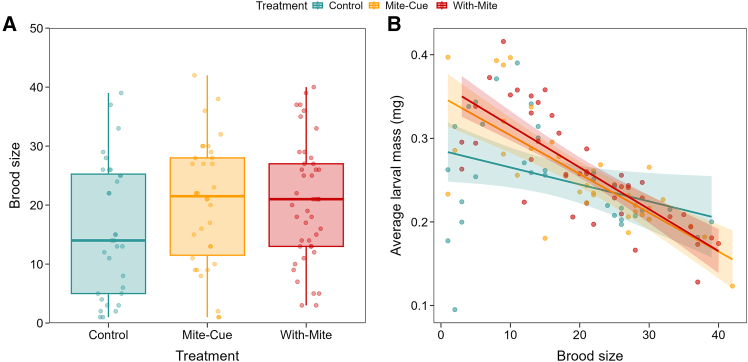
Mite association induces a shift in reproductive investment and alters the quantity-quality trade-off (A) Females in both the with-mite and mite-cue treatments produced significantly larger broods compared to the control group, consistent with a terminal investment-like strategy in response to perceived competition. Boxplots indicate the median (center line), interquartile range (box limits), and 1.5 interquartile range (whiskers). White circles represent group means, and colored points show individual breeding events. (B) The trade-off between offspring quantity (brood size) and quality (average larval mass). Lines represent linear regressions with 95% confidence intervals (shaded areas).

**Figure 3:**
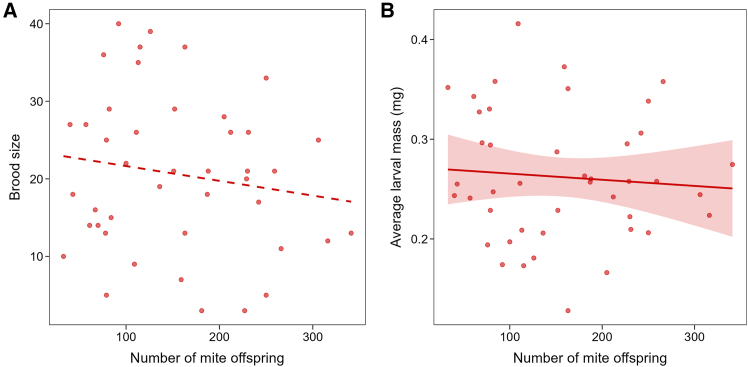
Effect of the number of mite offspring on beetle reproductive performance within the with-mite treatment (A) Beetle brood size was not significantly associated with the number of mite offspring present on the carcass. (B) Average larval mass shows a weak negative relationship with mite offspring number, suggesting a minor competitive effect of mite density on larval growth, though the effect size was negligible. Dashed and solid lines represent statistically non-significant and significant relationships from GLMMs, respectively. The shaded area presents a 95% confidence interval.

Mite treatment significantly influenced beetle reproductive outcomes (*χ*^2^ = 35.09, d.f. = 2, *p* < 0.001; Figure 2A). Females exposed to physical mites produced significantly larger broods compared to the no-mite control group (*z* = −5.10, *p* < 0.001). Interestingly, females in the mite-cue treatment (where mites were physically present for 24 h but removed prior to breeding) adopted a similar high-fecundity strategy, producing significantly more offspring than controls (*z* = −5.38, *p* < 0.001) and matching the brood sizes of the with-mite treatment (z = 0.67, *p* = 0.781). Brood size was positively predicted by clutch size (*χ*^2^ = 86.72, d.f. = 1, *p* < 0.001; Figure S1), a relationship that held consistently across all treatments (treatment x clutch size: *χ*^2^ = 4.07, d.f. = 2, *p* = 0.131). Within the with-mite treatment, the number of mite offspring did not significantly explain variation in beetle brood size (*χ*^2^ = 0.34, d.f. = 1, *p* = 0.560; Figure 3A).

### Context-dependent trade-offs in offspring quality

Offspring quality, measured as average larval mass, was also affected by mite treatment, but this effect was strongly conditional on brood size (treatment × brood size: *χ*^2^ = 8.82, d.f. = 2, *p* = 0.012; Figure 2B). As expected, larger broods generally resulted in smaller individual larvae in the no-mite control group (*χ*^2^ = 7.17, d.f. = 1, *p* = 0.007), indicating a classic quality-quantity trade-off. Notably, this trade-off slope was significantly steeper for beetles in both the with-mite (*t* = 2.61, *p* = 0.028) and mite-cue (*t* = 2.41, *p* = 0.047) treatments compared to controls. This suggests that while mite-associated mothers produced more offspring, they did so at a steeper per-capita physiological cost to larvae. Additionally, the number of mite offspring in the with-mite group showed a weak but statistically negative relationship with average larval mass (*χ*^2^ = 5.46, d.f. = 1, *p* = 0.019; Figure 3B), although the effect was negligible (Estimate = −0.000188 ± 0.000080, *t* = −2.34).

### Physiological trajectories and body mass dynamics

Analysis of body mass trajectories revealed no significant main effect of treatment (*χ*^2^ = 0.02, d.f. = 2, *p* = 0.992), but physiological dynamics differed substantially across groups over time (treatment × stage: *χ*^2^ = 15.72, d.f. = 4, *p* = 0.003; Figure S2). All females, regardless of treatment, gained mass significantly during the carcass preparation phase (initial vs. post-preparation: all *p* < 0.001; Figure S2), confirming uniform nutritional intake prior to larval hatching. However, the subsequent cost of reproduction in terms of body mass diverged. Mothers in the control and with-mite treatments exhibited similar, steep physiological declines during the brood care phase, resulting in substantial net body mass losses by the time of larval dispersal (Δnet loss: control = 0.020 ± 0.003 g; with-mite = 0.026 ± 0.003 g). In contrast, females exposed only to mite cues followed a shallower trajectory. Despite rearing enlarged broods, mite-cue mothers retained a body mass significantly closer to their initial state (Δnet loss = 0.010 ± 0.003 g), effectively halving the body mass loss observed in the other groups.

**Figure 4:**
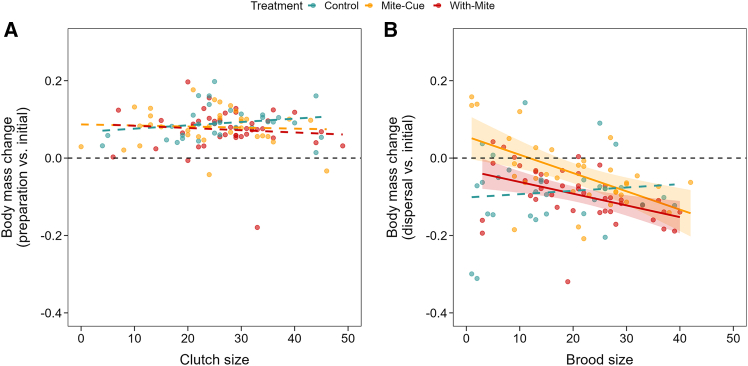
Context-dependent somatic costs of reproduction (A) Proportional change in body mass during the carcass preparation and egg-laying phase (i.e., initial to carcass preparation on Day 5) as a function of clutch size. (B) Proportional change in body mass at the dispersal stage (i.e., initial to dispersal) as a function of brood size. Dashed and solid lines represent non-significant and statistically significant relationships from GLMMs, respectively. The shaded area presents a 95% confidence interval.

This divergence was further clarified by analyzing proportional mass change as a function of reproductive effort. During the initial carcass preparation and egg-laying phase, proportional body mass change was not significantly affected by treatment (*χ*^2^ = 2.63, *p* = 0.269) or clutch size (*χ*^2^ = 0.09, *p* = 0.767; Figure 4A), indicating that the energetic cost of manipulating the carcass and egg laying was uniform across groups. However, following the brood care phase, the proportion of mass loss varied significantly with brood size dependent upon treatment (treatment × brood size: *χ*^2^ = 11.06, d.f. = 2, *p* = 0.004; Figure 4B). Control females incurred a fixed mass loss, losing body mass regardless of brood size (*χ*^2^ = 0.24, d.f. = 1, *p* = 0.627). In contrast, beetles breeding with mites exhibited a variable cost, where mass loss increased significantly with brood size (*χ*^2^ = 10.08, d.f. = 1, *p* = 0.001). Mite-cue females showed a similar pattern, with mass loss increasing significantly with brood size (χ^2^ = 14.70, d.f. = 1, *p* < 0.001), yet they maintained a significantly higher post-reproductive body condition compared to both control and with-mite females (vs. control: *t* = −2.77, *p* = 0.019; vs. with-mite: *t* = 3.00, *p* = 0.010). This suggests that mite-cue females paid a brood-size-dependent mass loss, but did so more efficiently than females physically burdened by mites.

### Survival consequences of reproductive effort

**Figure 5:**
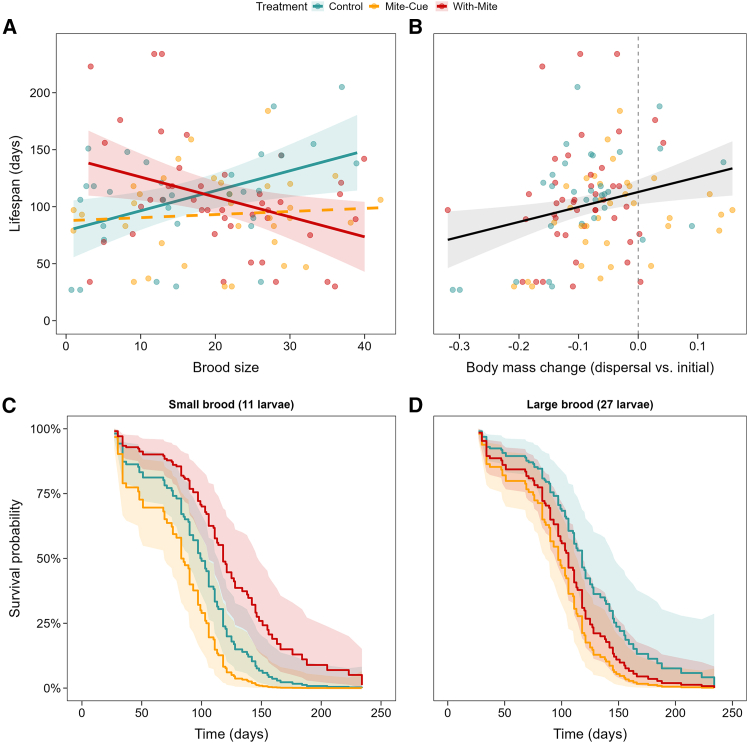
Mite association alters the survival costs of reproduction (A) Context-dependent survival trade-offs, with brood size differently predicting the survival costs. (B) The relationship between brood size and lifespan by varied treatments, showing greater proportional mass loss during breeding, was significantly associated with shorter maternal lifespans. The solid black line represents the statistically significant universal trend across all groups. Dashed and solid lines represent non-significant and statistically significant relationships from GLMMs, respectively. The shaded area presents a 95% confidence interval. (C and D) Predicted survival curves from the cox proportional hazards model, evaluated at small (11 larvae; 25th percentile) and large (27 larvae; 75th percentile) brood sizes, with proportional body mass change held at its mean. Shaded ribbons indicate 95% confidence intervals. At low reproductive effort, survival trajectories were broadly similar across treatments. With high reproductive effort, with-mite females experienced markedly accelerated mortality relative to control females, while mite-cue females showed intermediate survival, illustrating the context-dependent nature of the reproduction-survival trade-off.

Female lifespan was significantly predicted by treatment, brood size, and their interaction (Table 1). The proportional hazards assumption was satisfied for all terms except proportion of body mass change, which showed a marginal violation (body mass change: χ^2^ = 5.33, *p* = 0.021); however, the global test was non-significant (χ^2^ = 10.25, d.f. = 6, *p* = 0.115), indicating the model was acceptable overall. Slope contrasts revealed that in the with-mite treatment, larger broods were associated with increased mortality risk (β = 0.453, 95% CI [0.019, 0.887]; Figure 5A), whereas in the control treatment, larger broods were associated with reduced mortality risk (β = −0.572, 95% CI [−0.981, −0.163]). The mite-cue treatment showed an intermediate, non-significant negative slope (β = −0.435, 95% CI [−0.944, 0.074]). Pairwise slope contrasts confirmed that the with-mite slope differed significantly from both control (*z* = −3.35, *p* = 0.002) and mite-cue (*z* = −2.71, *p* = 0.019), while control and mite-cue did not differ from each other (*z* = −0.44, *p* = 0.901). Independent of the interaction, brood size (χ^2^ = 7.52, d.f. = 1, *p* = 0.006) and maternal mass change following the brood care phase (χ^2^ = 7.79, d.f. = 1, *p* = 0.005; Figure 5B) each significantly predicted lifespan, with greater mass loss associated with reduced survival (Figures 5C and 5D).

**Table 1:** Mixed-effects cox proportional hazards model of maternal lifespan

Source				
Mixed-effects cox proportional hazards model				
	df	χ^2^	*P*	
Treatment	2	10.85	0.004	
Brood size	1	7.52	0.006	
Maternal mass change	1	7.79	0.005	
Treatment × brood size	2	12.55	0.002	
Slope contrasts: brood size effect by treatment (emtrends)				
Treatment	*β*	95% CI	SE	
Control	−0.572	[−0.981, −0.163]	0.209	
Mite-cue	−0.435	[−0.944, 0.074]	0.260	
With-mite	0.453	[0.019, 0.887]	0.221	
Pairwise slope contrasts (Tukey-adjusted)				
Contrast	*Δβ*	SE	*z*	*P*
Control v mite-cue	−0.137	0.314	−0.44	0.901
Control v with-mite	−1.025	0.306	−3.35	0.002
Mite-cue v with-mite	−0.888	0.328	−2.71	0.019
Proportional hazards assumption (Schoenfeld residuals)				
Term	df	χ^2^	*P*	
Treatment	*2*	2.42	0.298	
Brood size	1	0.97	0.325	
Maternal mass change	1	5.33	0.021	
Treatment × brood size	2	1.45	0.484	
Global	6	10.25	0.115	

χ^2^ statistics are Type III Wald tests. Slope contrasts (emtrends) show the effect of brood size on the log-hazard of death within each treatment, with pairwise Tukey-adjusted comparisons. Proportional hazards assumption was tested using Schoenfeld residuals on a fixed-effects equivalent (coxph); the global test confirms no violation.

## Discussion

Life-history theory posits that organisms must allocate finite resources between current reproduction and somatic maintenance, creating fundamental trade-offs that dictate fitness.^36^^,^^37^ The expression of these trade-offs is context-dependent, and we predicted that the distinction between a symbiont’s physical burden and the perceived risk of competition it imposes would shape reproductive decisions along two axes: triggering anticipatory escalation of reproductive investment and modulating the somatic cost paid for that escalation Our results support this framework but reveal a more nuanced picture, in which mite-associated cues shape not only the magnitude but also the completion of reproductive attempts.

A central observation is that mite-associated cues influenced not whether beetles attempted to reproduce, but how successfully they completed it. Egg-laying rates were broadly similar across treatments, yet the proportion of females successfully producing dispersing larvae diverged considerably; with-mite-associated females were more likely to produce a brood than controls. Because our reproductive success metric was binary, this divergence cannot reflect differential brood culling; rather, it points to whole-clutch failures arising from carcass degradation, egg failure, or maternal abandonment. Several lines of evidence argue against a straightforward competitor framing of mites in this system: brood size was larger under mite presence, mite offspring numbers did not predict beetle brood size, and only a weak negative association emerged between mite numbers and larval mass. Instead, we suggest that live mite presence functions partly as a signal of carcass viability and/or local competitor density, sustaining maternal commitment throughout larval development.^32^ Mite-cue females, receiving only a one-time signal, initiated breeding at rates comparable to with-mite females but were no more likely than controls to complete it, consistent with cues triggering the threshold for breeding without sustaining the ongoing investment required to convert eggs into dispersing larvae.

### Non-consumptive cues drive reproductive escalation

In coevolved phoretic associations, it is often assumed that the fitness costs to the host are minimal because the relationship is typically commensal or nearly so.^33^ For example, mites have evolved to limit detrimental effects, and hosts in turn tolerate or sometimes even benefit under specific conditions. This suggests a balance achieved through selection to minimize fitness costs while preserving the association.^28^ Our findings challenge this assumption: non-consumptive effects alone were sufficient to trigger substantial shifts in reproductive strategy. Mite-cue females adopted a high-fecundity strategy indistinguishable from that of with-mite females, despite having no physical contact with mites during breeding itself. Because mites were removed prior to breeding in the mite-cue treatment, this response cannot be attributed to direct exploitation or energetic drain imposed by mites. Instead, we suggest that *N. nepalensis* uses the brief physical sensation of phoretic mites during carcass discovery as an anticipatory cue, informing the female both about local competitor density (and therefore the uncertainty of future reproductive opportunities) and about current carcass viability. Brief exposure to conspecific cues during development is sufficient to trigger anticipatory reproductive investment in other taxa. For instance, male field crickets (*Teleogryllus oceanicus*) exposed to adult male song during nymphal stages invested more in reproductive tissue. Similarly, bank voles (*Myodes glareolus*) exposed to the odor of rival males developed larger accessory glands.^38^

Overall, both interpretations predict the observed reproductive escalation, and both are consistent with anticipatory plasticity, whereby organisms adjust life-history allocation based on perceived rather than realized costs.^39^ Our findings therefore extend the non-consumptive effects framework beyond predator-prey systems, demonstrating that subtle symbiont-associated cues can reshape host life-history allocation through perceptual pathways alone.

### Context-dependent trade-offs in offspring quality

While the cue-driven shift to larger broods suggests an adaptive attempt to maximize output, it incurred a more complex penalty on offspring quality than a simple quality-quantity trade-off. All treatments exhibited a negative relationship between brood size and average larval mass, but the slope was significantly steeper in the mite-exposed groups (with-mite and mite-cue) compared to controls (Figure 2B). Importantly, this steeper slope did not translate to uniformly poorer offspring under mite exposure: mite-associated mothers produced larvae of higher individual mass than controls at low-to-intermediate brood sizes (up to approximately 25 larvae), with offspring quality only declining below control levels at larger brood sizes. This pattern indicates that “reproductive escalation” under mite cues is not simply a shift toward quantity at the expense of quality; rather, mothers initially over-provision per offspring, perhaps to enhance offspring competitiveness in a perceived competition-rich environment. The finite carcass resource imposes a ceiling: Beyond approximately 25 larvae, density effects outweigh the benefits of increased maternal effort, and offspring quality declines sharply. The crossing of these reaction norms reframes the classic quality-quantity trade-off as a context-dependent reaction norm, with mite cues increasing both the maximum per-capita investment and the curvature of allocation across brood sizes.

Within the with-mite treatment, mite offspring numbers showed a weak but detectable negative association with average larval mass (Figure 3B), confirming that direct mite-larva resource competition does occur, though its effect is minor relative to the strategic shifts induced by mite exposure. This resolves a conflict in the literature: While mites may provide mutualistic benefits by suppressing blowflies in the wild (Sun and Kilner 2020), our results confirm that in the absence of interspecific competitors, they impose a modest direct cost on larval provisioning. The variable mite effects on beetle reproduction reported elsewhere^31^ likely reflect this context-dependence, with the net outcome shaped by the interaction between the host’s cue-driven strategy and the realized resource environment.

### Physiological costs and survival consequences

The divergence in maternal outcomes between with-mite and mite-cue females highlights the critical distinction between cue-driven plasticity and consumptive constraint. The reproductive escalation observed in both mite-exposed treatments is consistent with terminal investment, allocating available energy toward maximizing immediate output at the expense of future survival.^35^ However, the physiological feasibility of this strategy differed sharply by context. Mite-cue females, having responded to the anticipatory cues but escaped the physical burden, were able to sustain high fecundity without severe somatic depletion (Figure 4B). This implies that perceived risk influences allocation decisions, but without the energetic burden of the symbiont, the host can buffer the cost. In contrast, females breeding with mites faced an inescapable physiological cost. Physical mite association overrode the benefits of high individual quality, enforcing a direct trade-off between current reproduction and future survival. In control females, higher reproductive effort was associated with reduced mortality risk (consistent with high-quality individuals masking trade-offs). In the with-mite group, this relationship was reversed: Increasing brood size was associated with sharply elevated mortality risk. Mechanistically, our data identify somatic depletion as the universal threshold for mortality. Across all treatments, somatic condition at dispersal was a strong predictor of lifespan. Mites, therefore, do not alter the physiological threshold itself but drive females closer to or beyond it through the combined demands of elevated reproductive effort and direct resource extraction. In this sense, mites unmask a latent trade-off between reproduction and longevity that remains hidden in benign environments where individual quality dominates the relationship.

Our study advances understanding of the complex interplay between direct consumptive effects and non-consumptive effects in host-symbiont systems. By identifying somatic exhaustion as the mechanistic link between reproductive effort and survival, we provide a unifying explanation for how cues shape reproductive strategy, while realized symbiont pressure determines whether that strategy is sustainable. Our results emphasize that symbiotic partners are not merely passive riders but active drivers of host life-history evolution, capable of exerting selection pressures comparable to those of predators. Future work should investigate the proximate mechanisms mediating this response, including hormonal regulation and metabolic allocation pathways. Examining the long-term consequences for offspring survival, growth, and reproductive success will clarify whether this cue-induced plasticity represents an adaptive strategy or imposes hidden, transgenerational fitness costs.

### Limitations of the study

Several limitations should be considered when interpreting our findings. Our experimental design isolates the effect of prior mite exposure but does not identify the proximate sensory mechanism through which females detect mites; phoretic mites could be perceived through tactile, chemical, or vibrational cues during attachment, and disentangling these possibilities—for example, by presenting isolated mite-derived chemical extracts without physical contact—remains an important direction for future work. Our interpretation of mite presence as an anticipatory cue is therefore functional rather than mechanistic, and the dual possibility that mites signal both local competitor density and carcass viability could not be experimentally separated within the present design, since both covary naturally with mite load. Finally, our experiments were conducted under controlled laboratory conditions with a standardized carcass size, mite load, and the absence of interspecific competitors such as blowflies; because the mite-beetle interaction is strongly context-dependent, the magnitude and even the direction of these effects may differ under natural conditions where competitors, variable resources, and fluctuating mite densities co-occur.

## Resource availability

### Lead contact

Requests for further information should be directed to and will be fulfilled by the lead contact, Syuan-Jyun Sun (sjs243@ntu.edu.tw).

### Materials availability

This study did not generate new unique reagents.

### Data and code availability

• All original data for this work are available at Zenodo (https://doi.org/10.5281/zenodo.18365968).
• The code associated with data analyses for this work is also available at Zenodo (https://doi.org/10.5281/zenodo.18365968).
• Any additional information required to reanalyze the data reported in this paper is available from the lead contact upon request.

## Acknowledgments

We thank the editor and two anonymous reviewers whose constructive comments substantially improved this manuscript. We also thank the burying beetles and mice that made this research possible. We thank members of the Sun Lab, particularly Yi-Ta Wu, for mite colony maintenance. We also thank Benjamin Jarrett for his valuable advice on statistical analysis of lifespan data. Furthermore, we would like to thank Megan M.Y. Chang for providing the illustrations and designing the graphical abstract. S.-J.S. was supported by the NTU New Faculty Founding Research Grant, National Science and Technology Council 2030 Cross-Generation Young Scholars Program (112-2628-B-002-013-; 113-2628-B-002-028-; 114-2628-B-002-027-), Academic Research-Career Development Project (Sprout Research Projects; 115L7845) provided by National Taiwan University, and the Yushan Fellow Program (MOE-111-YSFAG-0003-002-P1) provided by the Ministry of Education.

## Author contributions

S.-J.S. conceived the study. All authors collected the data. S.-J.S. processed and analyzed the data. M.R., T.M., and S.-J.S. drafted the manuscript, and all authors contributed to later versions.

## Declaration of interests

The authors declare no competing interests.

## Star★Methods

### Key resources table

**Table:** 

RESOURCE	SOURCE	IDENTIFIER
**Biological samples**		

Beetle	Malik et al. 2025^40^	NA

**Deposited data**		

Raw data and code for analyses	This paper	Zenodo:
Mite	Lan et al. 2025^41^	NA

**Softwares and algorithms**		

R version 4.5.2	R Core Team 2024^42^	https://www.R-project.org/

**Other**		

Analytical Balances	https://www.shimadzu.com/an/products/balances/analytical-balances/at-r-series/spec.html	Shimadzu ATX224R

### Experimental model and study participant details

#### Burying beetle laboratory colony

We used laboratory-reared burying beetles Nicrophorus nepalensis, originating from field-collected adults in northern Taiwan (see Malik et al.^40^ for detailed field collection protocols). To maintain genetic diversity and prevent inbreeding, approximately 80 wild-caught pairs were introduced into the laboratory colony annually during the breeding season (March–May). Beetles were maintained under controlled environmental conditions that mimicked natural daily fluctuations: a mean temperature of 17.8°C with the daily temperature fluctuation (16°C–20°C) under a 10:14 light-to-dark cycle, 70% relative humidity. Individual adults were housed in plastic containers and fed twice a week with pork mince until used in experiments.

#### Phoretic mite laboratory colony

The phoretic mite colony (*Poecilochirus carabi*) was established from deutonymphs collected from wild N. nepalensis of the same population across six sites in northern Taiwan (Datun Natural Park, Fuzhou Shan Park, Xianjiyan, Luku Incident Memorial Park, Huangdidian, and Lishan) in 2022.^41^ Breeding colonies were maintained in large containers (14.2 cm diameter × 6.3 cm height) filled with 1.5 cm of moist soil. Each container was stocked with 20 mites and a sexually mature beetle pair provisioned with a mouse carcass (20–30 g), allowing mites to reproduce alongside their hosts. Upon larval dispersal, mites were harvested from parent beetles and transferred to fresh containers with new beetle hosts. Non-breeding mite populations were maintained on these vector beetles, which were fed twice a week with minced pork. To maintain the colony, we refreshed the population monthly by seeding new breeding containers (*n* = 10) with 20 mites selected from the most recent generation, ensuring a mix of individuals from different maternal lineages (see Lan et al. 2025 for further details on colony maintenance). Prior to the manipulative experiment, mites were carefully collected from colony beetles using fine brushes and tweezers.

### Method details

#### Experimental design

Across two experimental blocks, we established 56 mating pairs drawn from 42 male and 41 female lineages (Block 1: 19 male × 17 female lineages, 21 pairs; Block 2: 33 male × 35 female lineages, 35 pairs). Within each block, three brothers from one male lineage were paired with three sisters from one unrelated female lineage, with each of the three resulting pairs assigned to a different treatment, ensuring genetic equivalence across treatments.

To explicitly disentangle the direct consumptive effects of symbionts from their non-consumptive (risk-induced) effects, we employed a manipulative experiment using a split-brood block design. This design was chosen to strictly control for genetic background and maternal effects, ensuring that phenotypic differences among treatments reflected plasticity rather than intrinsic variation. We established genetically standardised mating blocks by selecting unrelated male and female families from our laboratory stock. Within each block, three brothers from a specific male lineage were paired with three sisters from a specific unrelated female lineage. These three genetically equivalent pairs were then distributed across three treatments (Figure 1) (*n* = 74, 73, 79 for control, mite-cue, and with-mite, respectively): (1) Control: Beetles were never exposed to *P. carabi* mites, serving as a baseline for reproductive performance in the absence of symbiont; (2) Mite-cue: Beetles were inoculated with 10 phoretic mites one day prior to breeding to allow for physical attachment and interaction. Crucially, however, all mites were experimentally removed immediately prior to carcass provision. This treatment was designed to isolate non-consumptive effects - physiological or behavioral changes driven by the prior perception of mite presence - in the absence of any direct physical burden during the actual breeding attempt; and (3) With-mite: Beetles were experimentally inoculated with 10 phoretic mites, identically to the mite-cue treatment, but these mites remained associated throughout the entire reproductive cycle, representing the combined direct and non-consumptive effects of symbiosis. This standardised load corresponds to the mean infestation intensity observed in natural populations (approximately 10 mites per beetle,^29^^,^^41^ ensuring that our treatment reflects the typical ecological context of this interaction rather than an artificial extreme.

#### Experimental procedure and mite manipulation

Experiments were conducted under standardised laboratory conditions (17.8°C, 16°C–20°C daily fluctuation, 10:14 h light:dark cycle). Sexually mature pairs (14 days post-eclosion) were placed in breeding containers (14.2 × 6.3 cm) filled with 2 cm of moist soil and provided with a standardised, freshly thawed mouse carcass (23–29 g). Mite manipulations followed a strict timeline: for with-mite and mite-cue treatments, females were inoculated with mites 24 h prior to carcass introduction (Day −1). On Day 0, immediately before the pair was introduced to the carcass, mites were carefully removed from mite-cue females using fine forceps, while mites on with-mite females were left undisturbed. Carcass preparation and egg laying typically concluded within 90 h. At this post-preparation stage (Day 4), we recorded clutch size by counting all eggs visible at the bottom of the container, a method previously validated to accurately estimate reproductive input.^43^ Larval development proceeded until dispersal (approximately Day 12), at which point we recorded brood size (number of surviving larvae) and total brood mass. Average larval mass was calculated as total brood mass divided by brood size, serving as a metric for offspring quality.

#### Physiological measurements and survival monitoring

To assess the associated costs of reproduction, female body mass was measured to the nearest 0.0001 g (Shimadzu ATX224R) at three critical physiological stages: (1) Initial mass: recorded on Day −1 prior to mite inoculation and breeding; (2) post-preparation mass: recorded on Day 4 after carcass burial but prior to larval hatching; and (3) Final mass: recorded on Day 12 immediately after larval dispersal. Following the breeding event, females were transferred to individual plastic boxes (10.8 × 7.5 × 2.1 cm) with soil to monitor longevity. To ensure that survival differences reflected the costs of the preceding reproductive bout rather than ongoing parasitism, all mites were removed from with-mite females at the time of dispersal using CO_2_ anesthesia. Females in the control and mite-cue treatments underwent a sham CO_2_ procedure to control for handling effects. Survival was monitored twice weekly, with beetles provided *ad libitum* food (1.5 g minced pork) until death. Lifespan was calculated as the number of days that beetles survived post-dispersal, quantifying the long-term fitness consequences of the reproductive strategy adopted under different mite contexts.

### Quantification and statistical analysis

All analyses were performed using R version 4.5.2.^42^ Prior to statistical modeling, data were filtered to exclude failed breeding attempts (females that produced no surviving larvae). Reproductive success was assessed in two steps: we first tested whether egg-laying rates differed among treatments using a Pearson chi-square test, and second, among females that successfully laid eggs, whether the probability of rearing dispersing larvae differed among treatments using a Pearson chi-square test followed by pairwise Fisher’s exact tests with Bonferroni correction.^44^^,^^45^

We fitted generalised linear mixed models (GLMMs) using the package *lme4*.^46^ To account for the split-brood experimental design and non-independence of siblings, all models included the identity of the sibling group (hereafter “pair identity”) nested within the block as a random factor, unless otherwise stated. Statistical significance of fixed effects was determined using Type III Wald χ2 tests (package *car*^47^). When significant interactions or main effects were detected, we performed post-hoc pairwise comparisons using estimated marginal means (package *emmeans*^48^) with *p*-values Tukey-adjusted for multiple comparisons.

Brood size (number of dispersing larvae) across treatments was modeled using a negative binomial GLMM to account for overdispersion, with treatment as the fixed effect and carcass mass as a covariate. Average larval mass was modeled using a Gaussian GLMM with treatment, brood size, and their interaction as fixed effects, and carcass mass as a covariate. To formally characterise the form of the treatment × brood size interaction on offspring quality, we conducted a simple slopes analysis using the *emmeans* package,^48^ extracting the within-treatment slope of the brood size–average larval mass relationship for each treatment group and performing pairwise slope contrasts (Tukey-adjusted).

Brood size and total brood mass were highly correlated in our dataset (r = 0.91, *p* < 0.001). We compared these as alternative predictors of reproductive effort in all downstream analyses using AIC. Brood size provided substantially better fit than total brood mass for average larval mass (ΔAIC = 49.64) and better fit for maternal proportional body mass change (ΔAIC = 3.28); the two metrics yielded equivalent fit in the Cox survival model (see later in discussion; ΔAIC = 0.36, well below the conventional threshold of 2). Full model comparison results are reported in Table S1. Brood size was therefore retained as the primary predictor throughout, as it more directly reflects the number of offspring successfully reared to dispersal—the fitness metric most relevant to life-history trade-off theory.

We analyzed maternal body mass dynamics in two ways. First, to assess mass loss relative to reproductive effort, we modeled the proportional mass change at two distinct stages: (1) Preparation phase (initial vs. post-preparation) and (2) Dispersal phase (initial vs. dispersal). These were analyzed in separate GLMMs. For the preparation phase, we included the interaction between treatment and clutch size. For the dispersal phase, we included the interaction between treatment and brood size. Carcass mass was included as a covariate in both models. Second, to visualise absolute mass trajectories, we analyzed absolute body mass using a Gaussian GLMM including the interaction between treatment and breeding stage (initial, preparation, dispersal) as fixed effects, with carcass mass as a covariate.

To further investigate the direct impact of mite burden on beetle reproduction within the with-mite treatment, we modeled the effect of mite offspring number on both brood size and average larval mass, separately. Because pair identity is nested within block but both levels contain limited replication within the with-mite subgroup, we report robustness checks comparing GLMM and simple GLM (without random effects) in Table S2. Inferences were substantively consistent across both model structures.

We analyzed maternal lifespan (days since eclosion) using a mixed-effects Cox proportional hazards model (package *coxme*^49^), with pair identity nested within block as a random effect. The model included treatment, brood size, maternal mass change (initial vs. dispersal), and their interaction (treatment × brood size) as fixed effects. To validate the proportional hazards assumption, Schoenfeld residual tests were performed on a fixed-effects equivalent (coxph) using the package *survival*, as cox.zph is not compatible with coxme objects. We further conducted a simple slopes analysis on the Cox model to formally quantify the brood size–mortality hazard slope within each treatment group and to test pairwise contrasts between treatments. The global test confirmed no violation (χ^2^ = 10.25, d.f. = 6, *p* = 0.115; Table 1). Predicted survival curves were generated from the Cox model at the 25th (11 larvae) and 75th (27 larvae) percentiles of observed brood size, with all other covariates held at their mean, to illustrate the context-dependent survival costs of reproduction across treatments.
