# Supplementary material for: Symbionts unmask the latent trade-off between reproduction and survival
Source: iScience. 2026 Jul 1;29(7):116582. doi: 10.1016/j.isci.2026.116582 (PMC13378352; doi:10.1016/j.isci.2026.116582)
Supplement: Document S1. Figures S1 and S2, and Tables S1 and S2 [file mmc1.pdf]

iScience, Volume *29*

Supplemental information

Symbionts unmask the latent trade-off between reproduction and survival

Mariana Gabrielle Cangco Reyes, Tanzil G. Malik, Ke-An Hong, and Syuan-Jyun Sun

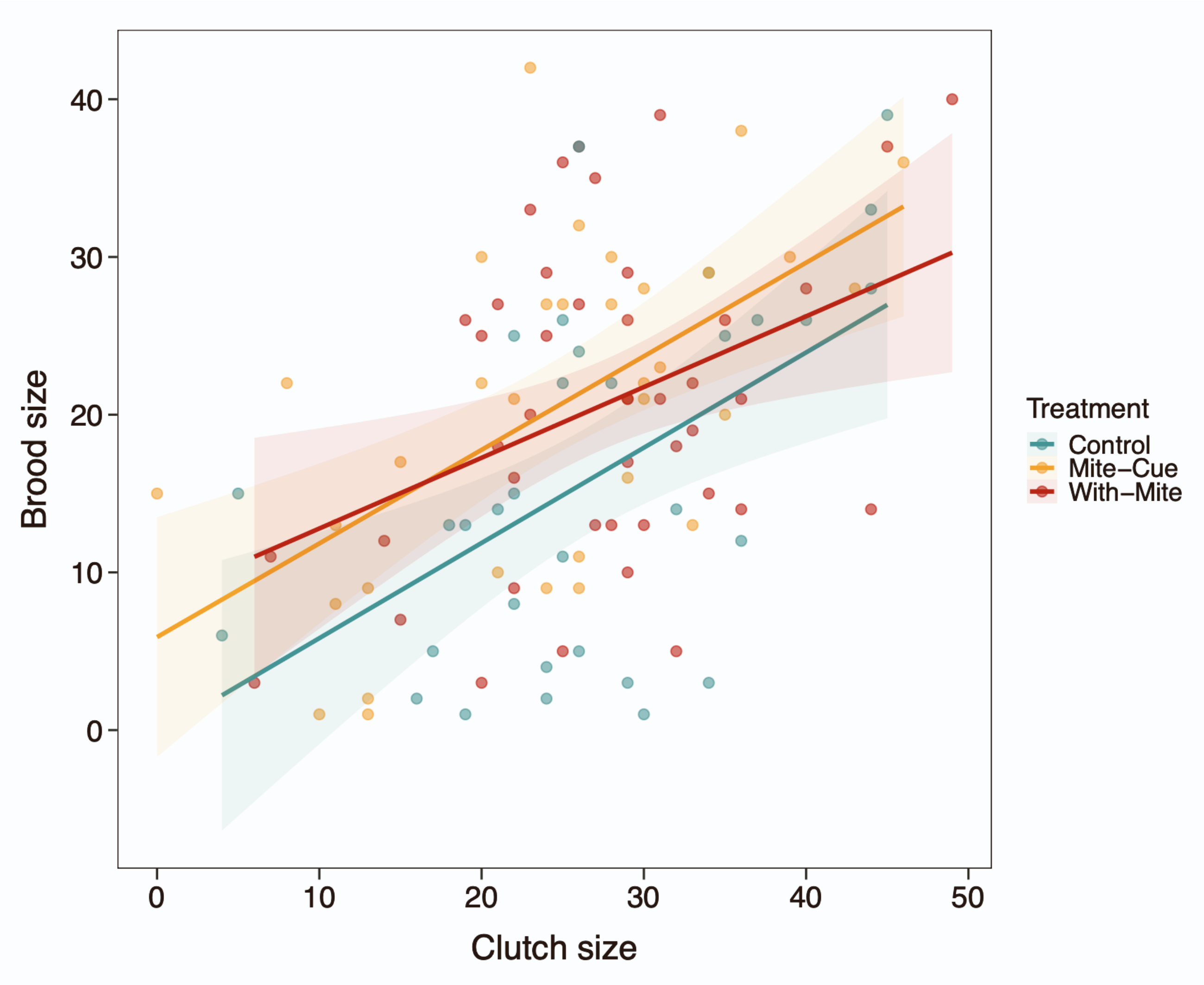

Figure S1. Clutch size positively predicts brood size independent of treatment, related to Figure 2. The lack of a significant treatment × clutch size interaction (*P* = 0.13) indicates that mite treatments did not alter the relationship between clutch size and brood size. Solid lines represent statistically significant relationships predicted from generalised linear mixed models (GLMMs); shaded areas represent 95% confidence intervals.

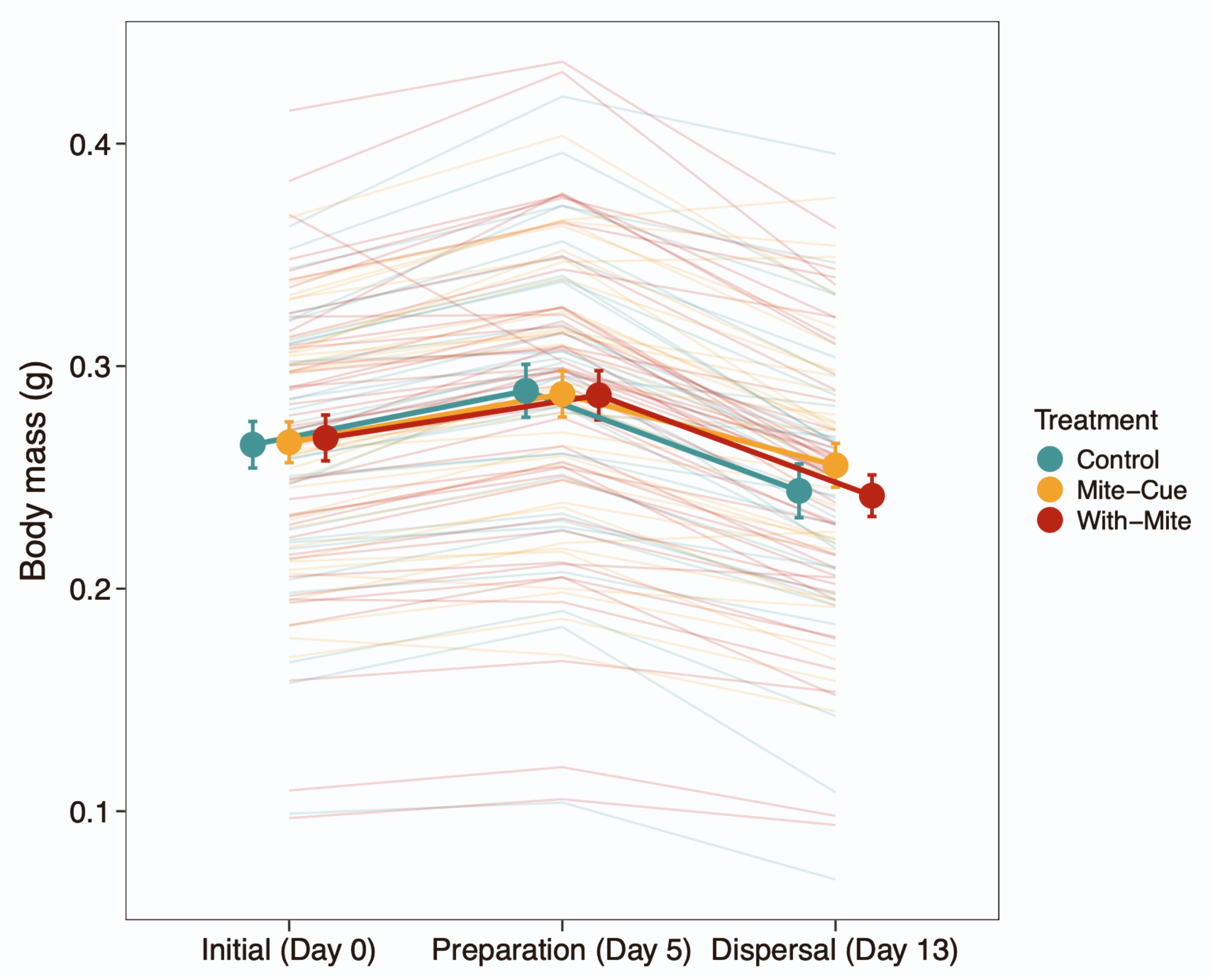

Figure S2. Temporal dynamics of maternal body mass across breeding, related to Figure 4. Body mass trajectories across three breeding stages: initial (Day 0), post-carcass preparation (Day 5), and dispersal (Day 13). Thin lines show individual females; thick points and error bars represent treatment means ± SEM.

Table S1. Comparison of models using brood size versus total brood mass as the predictor of reproductive effort and survival, related to STAR Methods. Models share an identical structure, differing only in whether reproductive effort is represented by brood size (number of dispersing larvae) or total brood mass. Lower AIC indicates better fit.

Predictor	n par.	AIC	BIC	logLik	−2 logLik	ΔAIC
**Average larval mass (GLMM)**						
Treatment × brood size + carcass mass + (1|block/pair)	10	−340.60	−313.68	180.30	−360.60	—
Treatment × total brood mass + carcass mass + (1|block/pair)	10	−290.96	−264.05	155.48	−310.96	49.64
ΔAIC = 49.64, brood size strongly preferred						
**Proportional body mass change (GLMM)**						
Treatment × brood size + carcass mass + (1|block/pair)	10	−237.78	−210.96	128.89	−257.78	—
Treatment × total brood mass + carcass mass + (1|block/pair)	10	−234.50	−207.67	127.25	−254.50	3.28
ΔAIC = 3.28, brood size preferred						
**Lifespan (mixed-effects Cox model)**						
Treatment × brood size + body mass change + (1|block/pair)	10	794.30	—	−387.15	774.30	0.36
Treatment × total brood mass + body mass change + (1|block/pair)	10	793.94	—	−386.97	773.94	—

ΔAIC = 0.36, equivalent fit (Δ < 2; no meaningful difference)

Models were compared by replacing brood size with total brood mass as the predictor of reproductive effort while retaining all other model terms. ΔAIC values are calculated as AIC(total brood mass model) − AIC(brood size model); positive values indicate that the brood size model is preferred. For the mixed-effects Cox model, AIC was computed manually as −2 × logLik + 2 × n par., as the coxme package does not directly report BIC. By the conventional thresholds for AIC differences (ΔAIC > 10 = decisive; ΔAIC > 4 = strong; ΔAIC > 2 = positive; ΔAIC < 2 = no meaningful difference), brood size is decisively preferred for average larval mass, positively preferred for proportional body mass change, and equivalent for lifespan. block = experimental block; pair = sibling-group identity nested within block.

Table S2. Comparison of models treating experimental block as a random effect versus a fixed effect, for the with-mite subgroup analyses, related to STAR Methods. χ^2 statistics are Type III Wald tests (with random effect) or likelihood-ratio tests (without random effect). F-values (superscript R) are reported for the linear model fitted without random effects. Results are substantively consistent across both model structures.

Term	***With random effect***			***Without random effect***	
	**χ^2**	**df**	***P***	**χ^2 / F**	***P***
**Model 1: Brood size ~ mite number (negative binomial)**					
Mite offspring number	0.34	1	*0.560*	0.28	*0.598*
Clutch size	6.80	1	*0.009*	6.74	*0.009*
Carcass mass	0.90	1	*0.342*	0.89	*0.346*
Block (fixed)	—	—	—	0.00	*0.996*
**Model 2: Average larval mass ~ mite number (linear)**					
Mite offspring number	4.66	1	*0.031*	6.27^{R}	*0.017*
Brood size	82.51	1	*<0.001*	75.68^{R}	*<0.001*
Carcass mass	0.25	1	*0.615*	0.51^{R}	*0.481*
Block (fixed)	—	—	—	2.99^{R}	*0.092*

In models with random effects, block was included as a random intercept; in models without random effects, block was instead included as a fixed effect. Superscript R denotes an F-value from the linear model. Across both formulations, the effect of mite offspring number on brood size remained non-significant, and its weak negative effect on average larval mass remained significant, confirming that the inferences are robust to the treatment of block.
